# Supplementary material for: Association of multiple tumor markers with newly diagnosed gastric cancer patients: a retrospective study
Source: PeerJ. 2022 May 19;10:e13488. doi: 10.7717/peerj.13488 (PMC9124459; doi:10.7717/peerj.13488)
Supplement: Supplemental Information 1 [file peerj-10-13488-s001.docx]

STROBE Statement—checklist of items that should be included in reports of observational studies

|  | Item No. | Recommendation | Page  No. | Relevant text from manuscript |
| --- | --- | --- | --- | --- |
| **Title and abstract** | 1 | (*a*) Indicate the study’s design with a commonly used term in the title or the abstract | 1 | A retrospective study |
|  |  | (*b*) Provide in the abstract an informative and balanced summary of what was done and what was found | 1 | Our research shows that gastric cancer is associated with age, gender, and the positive levels of AFP, CEA, CA125, CA199, and CA242. |
| Introduction | | | |  |
| Background/rationale | 2 | Explain the scientific background and rationale for the investigation being reported | 2 | Although the correlation between gastric cancer and eight tumor markers has been extensively studied, the data on positive rates of tumor markers and gastric cancer in Sichuan are very limited, and there has been no systematic evaluation of these eight markers on the same cohort using the Cochran–Mantel–Haenszel (CMH) test or logistic regression analysis |
| Objectives | 3 | State specific objectives, including any prespecified hypotheses | 3 | The objectives of this paper were to study the relationship between newly diagnosed gastric cancer and tumor markers using a commercial multi-tumor marker detection kit |
| Methods | | | |  |
| Study design | 4 | Present key elements of study design early in the paper | 3-4 | newly diagnosed gastric cancer patients, The determination of the gastric cancer and control group sample sizes, CMH test, logistic regression analysis |
| Setting | 5 | Describe the setting, locations, and relevant dates, including periods of recruitment, exposure, follow-up, and data collection | 3-4 | A total of 268 newly diagnosed gastric cancer patients (aged 60.91±11.51) who were admitted to Sichuan Cancer Hospital between June 2018 and December 2019 were selected. |
| Participants | 6 | (*a*) *Cohort study*—Give the eligibility criteria, and the sources and methods of selection of participants. Describe methods of follow-up  *Case-control study*—Give the eligibility criteria, and the sources and methods of case ascertainment and control selection. Give the rationale for the choice of cases and controls  *Cross-sectional study*—Give the eligibility criteria, and the sources and methods of selection of participants | 3-4 | The inclusion criteria was as follows: 1) Gastric cancer group: in line with the Guidelines for the Standardized Diagnosis and Treatment of Gastric Cancer (China, 2013) from the Chinese Medical Association, M stage was collected according to the AJCC 7th edition. 2) Control group: 209 subjects who underwent physical examination in this hospital during the same period were selected as the control group. Healthy physical examination: no serious heart, brain, liver, lung, kidney, or other primary diseases in the past, and relevant examinations were within the normal range; age> 18 years. Exclusion criteria: accompanied by major organ dysfunction, septic shock, hemorrhagic shock, myocardial infarction, benign tumors, any cancer or cancer history, recent hospitalizations or other diseases, pregnant or lactating, excluded various factors that may have had an effect on the tumor markers. |
|  |  | (*b*) *Cohort study*—For matched studies, give matching criteria and number of exposed and unexposed  *Case-control study*—For matched studies, give matching criteria and the number of controls per case | 3-4 | Guidelines for the Standardized Diagnosis and Treatment of Gastric Cancer (China, 2013), 268 newly diagnosed gastric cancer patients, CLSI (Horowitz, 2008) guidelines, 209 subjects who underwent physical examination in this hospital during the same period were selected as the control group |
| Variables | 7 | Clearly define all outcomes, exposures, predictors, potential confounders, and effect modifiers. Give diagnostic criteria, if applicable | 3-4 | in line with the Guidelines for the Standardized Diagnosis and Treatment of Gastric Cancer (China, 2013) from the Chinese Medical Association, all diagnoses were performed by gastroscopy, CT, or B-ultrasound, and gastric cancer was confirmed by gastroscopy or postoperative pathological biopsy; no other primary cancer sites; no history of chemotherapy, radiotherapy, or immunotherapy |
| Data sources/ measurement | 8* | For each variable of interest, give sources of data and details of methods of assessment (measurement). Describe comparability of assessment methods if there is more than one group | *4* | *The multi-tumor marker detection kit (Chaozhou Shukang Biotechnology Co., Ltd.), LU-07 biochip reader (Shanghai Mingyuan Shukang Biochip Co., Ltd.), and biochip image analysis system software were obtained from Huzhou Shukang Biotechnology Co., Ltd.*  *We used a pyrogen-free and endotoxin test tube to draw 2 ml of fasting venous blood in the early morning the day after the patient's admission and excluded various factors that may have had an effect on the tumor markers. The serum samples were collected without hemolysis after centrifugation. After the serum was antiquated, it was stored in a refrigerator at 4°C, tested within 5 days, and equilibrated to room temperature before testing. We strictly followed the manufacturer’s instructions for the biochip reader.* |
| Bias | 9 | Describe any efforts to address potential sources of bias | 4 | After the normality test, the skewed distribution (non-normal distribution) was transformed using the logarithmic transformation. |
| Study size | 10 | Explain how the study size was arrived at | 4 | The determination of the gastric cancer (considering the sample size met the minimum of 165, α=0.01, β=0.05, Pt=0.2, Pc=0.05, Nt:Nc=1) and control group (considering the sample size met the minimum of 120) sample sizes were based upon the Sample Size Calculations in Clinical Research, Third Edition (Chow et al., 2017) and the CLSI (Horowitz, 2008) guidelines. |

Continued on next page

| Quantitative variables | 11 | Explain how quantitative variables were handled in the analyses. If applicable, describe which groupings were chosen and why | 4 | The distribution of the data of the eight individual gastric cancer biomarkers was analyzed using the one-sample Kolmogorov-Smirnov and Shapiro-Wilk tests. The analytical results determined whether the parametric or non-parametric statistical method would be used in the following analysis. After the normality test, the skewed distribution (non-normal distribution) was transformed using the logarithmic transformation. |
| --- | --- | --- | --- | --- |
| Statistical methods | 12 | (*a*) Describe all statistical methods, including those used to control for confounding | 4 | the one-sample Kolmogorov-Smirnov and Shapiro-Wilk tests, the logarithmic transformation, CMH test, Logistic regression analysis |
|  |  | (*b*) Describe any methods used to examine subgroups and interactions | 5 | CMH test |
|  |  | (*c*) Explain how missing data were addressed | 5 | Figure 1 |
|  |  | (*d*) *Cohort study*—If applicable, explain how loss to follow-up was addressed  *Case-control study*—If applicable, explain how matching of cases and controls was addressed  *Cross-sectional study*—If applicable, describe analytical methods taking account of sampling strategy | 3-4 | The determination of the gastric cancer (considering the sample size met the minimum of 165, α=0.01, β=0.05, Pt=0.2, Pc=0.05, Nt:Nc=1) and control group (considering the sample size met the minimum of 120) sample sizes |
|  |  | (*e*) Describe any sensitivity analyses | 4 | Logistic regression analysis was used to establish the diagnostic mathematical model. On the basis of this model, the prediction value was calculated, followed by ROC curve analysis |
| Results | | | | |
| Participants | 13* | (a) Report numbers of individuals at each stage of study—eg numbers potentially eligible, examined for eligibility, confirmed eligible, included in the study, completing follow-up, and analysed | 5 | Table3-Table5 |
|  |  | (b) Give reasons for non-participation at each stage | 5 | The main reasons for the exclusion of subjects |
|  |  | (c) Consider use of a flow diagram | 5 | Figure 1 |
| Descriptive data | 14* | (a) Give characteristics of study participants (eg demographic, clinical, social) and information on exposures and potential confounders | 5 | After excluding participants who did not meet the baseline, a total of 268 gastric cancer patients and 209 healthy people were included in the analysis. |
|  |  | (b) Indicate number of participants with missing data for each variable of interest | 5 | Figure 1 |
|  |  | (c) *Cohort study*—Summarise follow-up time (eg, average and total amount) | *N/A* | *N/A* |
| Outcome data | 15* | *Cohort study*—Report numbers of outcome events or summary measures over time | *N/A* | *N/A* |
|  |  | *Case-control study—*Report numbers in each exposure category, or summary measures of exposure | *5-6* | *Table3-Table5* |
|  |  | *Cross-sectional study—*Report numbers of outcome events or summary measures | *N/A* | *N/A* |
| Main results | 16 | (*a*) Give unadjusted estimates and, if applicable, confounder-adjusted estimates and their precision (eg, 95% confidence interval). Make clear which confounders were adjusted for and why they were included | 5-6 | Table3-Table5 |
|  |  | (*b*) Report category boundaries when continuous variables were categorized | 5 | Table 2 |
|  |  | (*c*) If relevant, consider translating estimates of relative risk into absolute risk for a meaningful time period | 5-6 | males were 3.329 times more likely to have gastric cancer than females. The over-54 years old age group had a risk of gastric cancer that was 6.342 times that of the under-54 years patients. the risk of CA125-positive patients being diagnosed with gastric cancer was 16.673 times that of CA125-negative patients |

Continued on next page

| Other analyses | 17 | Report other analyses done—eg analyses of subgroups and interactions, and sensitivity analyses | 5-6 | Sensitivity, specificity, and accuracy of single and combined tumor markers |
| --- | --- | --- | --- | --- |
| Discussion | | | | |
| Key results | 18 | Summarise key results with reference to study objectives | 9 | gastric cancer is associated with age, gender, and the positive levels of AFP, CEA, CA125, CA199, and CA242. The positive levels of AFP and CA125 are related to the distant metastasis of gastric cancer. Combined detection based on logistic regression analysis can be used for initial screening of gastric cancer to a certain extent |
| Limitations | 19 | Discuss limitations of the study, taking into account sources of potential bias or imprecision. Discuss both direction and magnitude of any potential bias | 9 | there were a few limitations to our study |
| Interpretation | 20 | Give a cautious overall interpretation of results considering objectives, limitations, multiplicity of analyses, results from similar studies, and other relevant evidence | 9 | Combined detection based on logistic regression analysis can be used for initial screening of gastric cancer to a certain extent. |
| Generalisability | 21 | Discuss the generalisability (external validity) of the study results | 10 | To improve the early diagnosis of gastric cancer and to reduce the missed diagnosis rate, higher-risk populations should first be identified through tumor marker detection, then imaging, gastroscopy, and colonoscopy. |
| Other information | |  | | |
| Funding | 22 | Give the source of funding and the role of the funders for the present study and, if applicable, for the original study on which the present article is based | N/A | N/A |

*Give information separately for cases and controls in case-control studies and, if applicable, for exposed and unexposed groups in cohort and cross-sectional studies.

**Note:** An Explanation and Elaboration article discusses each checklist item and gives methodological background and published examples of transparent reporting. The STROBE checklist is best used in conjunction with this article (freely available on the Web sites of PLoS Medicine at http://www.plosmedicine.org/, Annals of Internal Medicine at http://www.annals.org/, and Epidemiology at http://www.epidem.com/). Information on the STROBE Initiative is available at www.strobe-statement.org.
